# Uterine Artery Embolisation of Fibroids and the Phenomenon of Post-Embolisation Syndrome: A Systematic Review

**DOI:** 10.3390/diagnostics12122916

**Published:** 2022-11-23

**Authors:** Michael G. Waldron, Yara W. Kassamani, Alexander T. O’Mahony, Siobhain M. O’Mahony, Orfhlaith E. O’Sullivan, Stephen P. Power, Liam Spence, Michael M. Maher, Owen J. O’Connor, Maria M. Buckley

**Affiliations:** 1 Department of Radiology, University College Cork, T12 AK54 Cork, Ireland; 2 School of Medicine, University College Cork, T12 AK54 Cork, Ireland; 3 Department of Radiology, Cork University Hospital, T12 DFK4 Cork, Ireland; 4 Department of Anatomy, University College Cork, T12 XF62 Cork, Ireland; 5 Department of Obstetrics and Gynaecology, Cork University Hospital, T12 DFK4 Cork, Ireland; 6 APC Microbiome Ireland, University College Cork, T12 TP07 Cork, Ireland; 7 Department of Pharmacology and Therapeutics, University College Cork, T12 XF62 Cork, Ireland

**Keywords:** uterine artery embolization, post-embolisation syndrome, fibroids, inflammation

## Abstract

Post-embolisation syndrome (PES) is a prevalent complication that occurs in patients following uterine artery embolisation (UAE) for the treatment of uterine fibroids. The aetiology of PES remains incompletely understood, although postulated to result secondary to tissue infarction resulting in release of inflammatory mediators. We followed PRISMA guidelines and performed a systematic review of studies of PES following UAE from inception to October 2022. Our published protocol was prospectively registered. Our search yielded 54 results. We reviewed 22 full texts, and nine articles were included. Observational studies comprised 6/9 relevant studies, with 5/9 retrospective design. The rate of PES was documented in 5/8 studies (excluding case report) with a reported incidence ranging from 4–34.6%. Five of the nine studies studies postulated that the aetiological basis of PES is inflammatory related. Further research is necessary to advance our understanding of PES to define the biological basis of the syndrome with more certainty and gain a consensus on peri-procedure management to reduce incidence and improve patient outcomes.

## 1. Introduction

Uterine fibroids are the most prevalent benign gynaecological tumour [1]. The exact aetiology determining the maturation and regression of fibroids remains elusive, although their growth is known to be triggered by sex steroid hormones [2]. Fibroids are generally diagnosed in the later reproductive period, with a reported incidence of 70% in white women and in excess of 80% in black women [1], whilst they regress in post-menopausal women, likely due to the ovaries producing diminishing levels of oestrogen [2]. Symptomatic fibroids can have a detrimental negative impact on patient quality of life (QoL) including frequent pressure pain, subfertility and more commonly heavy or prolonged menstrual bleeding [1,3]. In the United Kingdom, the National Institute of Clinical Excellence have reported that over one million women pursue medical attention for heavy menstrual bleeding per annum [4], with costs (direct and indirect) for uterine fibroids estimated at 34.4 billons dollars per annum in the United States [1].

Treatment of uterine fibroids is generally reserved for women who are either symptomatic or those with large fibroids who are planning pregnancy [3]. To date, no medical treatment for fibroids has demonstrated prolonged efficacy with acceptable safety data [1]. Gonadotrophin-releasing hormone analogues are effective at reducing the size of fibroids, however, prolonged use is limited by their negative effect on bone mineral density [1]. Surgical management of symptomatic fibroids is common, with a reported 30% of annual hysterectomies in the United States performed which is the second most common indication [3], with major adverse sequelae occurring in an estimated 7% of post hysterectomy [5].

Uterine artery embolization (UAE) is a minimally invasive procedure, which was initially described in 1995 as a successful alternative to surgery and hormonal treatment for symptomatic uterine fibroids [6]. In UAE, the uterine arteries and their branches bilaterally are occluded with particulate emboli, with the aim of inducing ischaemic necrosis of the fibroids, without resultant permanent negative sequalae on the otherwise normal uterus [1]. UAE is effective in reducing the symptomatology of treated women, with a reported 69% of women undergoing a technical successful procedure (avoiding a hysterectomy) and comparable QoL to hysterectomy after 10 years [7]. However, concerns remain regarding potential complications including post-embolisation syndrome, post procedure pain, infection, premature ovarian failure and secondary amenorrhoea [7,8,9]. UAE is a uterine preserving procedure, thereby an attractive potential option for subfertile women with large fibroids [9].

Post-embolisation syndrome (PES) following UAE is an iatrogenic phenomenon, which usually commences in the first 24 to 48 h post-procedure and resolves spontaneously over 10–14 days [10]. It is characterised by pelvic pain, low grade fever, myalgia, mild leucocytosis and discharge [11]. Current treatment entails supportive measures with pain relief, antiemetics, anti-inflammatory medications and hydration. With effective symptom control, patients are usually discharged within 24 h [3]. Diagnosis can be difficult, often requiring extensive investigations before confirmation, in order to exclude more sinister differentials including sepsis [12]. Previous studies performed on a related phenomenon post-ablation syndrome following hepatic and renal ablation treatment reported an incidence of 33% (12/36), with significant pain and interference with QoL [13].

The aim of this systematic review is to assess the available literature regarding the burden and management of PES in women post UAE.

## 2. Materials and Methods

### 2.1. Selection Criteria

A systematic review was performed according to published guidelines from the Cochrane Collaboration [14] and is reported according to the PRISMA guidelines [15]. A study protocol (Supplementary Table S1) was developed to include original research articles presenting data related post-embolisation syndrome from uterine artery embolisation. Studies involving post-operative complications and their management were included. All published observational studies and randomized trials were included if they met the following criteria: contained original data, human subjects, printed in English language, and full text was available. No date restrictions were applied.

### 2.2. Search Strategy

A systematic search of the literature was performed MEDLINE (PubMed), Cochrane and Embase databases. The databases were searched from inception to October 2022. The search was performed using key terms (Post-embolisation syndrome*) AND ((Uterine artery [title/abstract]) OR (Uterine fibroid)) (Appendix A). Two reviewers (M.W. and OJOC) independently screened titles and abstracts using the Rayyan web application for systematic review screening [16]. Full texts were sourced for relevant articles and Zotero used as our reference manager. Inclusion criteria were assessed independently (M.W. and OJOC), and the final list was agreed by consensus. A manual search of the reference lists of identified articles was performed. The systematic review was performed in accordance with the pre-specified protocol, which was prospectively registered on PROSPERO (CRD42022365162), the international prospective register of systematic reviews.

### 2.3. Data Extraction

For each study, the following data were collected:

(a) Title, journal, year of publication, country of origin study
(b) Study design and data collection (prospective or retrospective)
(c) Inclusion, exclusion criteria
(d) Study population number and demographics
(e) Type and indication for intervention
(f) Outcome measures: aetiology, post-procedure complications and clinical outcomes

### 2.4. Quality Assessment

The Effective Public Health Practice Project (EPHPP) quality assessment tool was utilised to analysis quality of included studies [17]. Quality areas assessed included study design, bias and study blinding, confounder management, data collection methods, intervention integrity and analysis. The EPHPP tool was used to grade each component, with each study then categorised as strong, moderate, or weak quality.

## 3. Results

### 3.1. Study Selection

**Figure 1:**
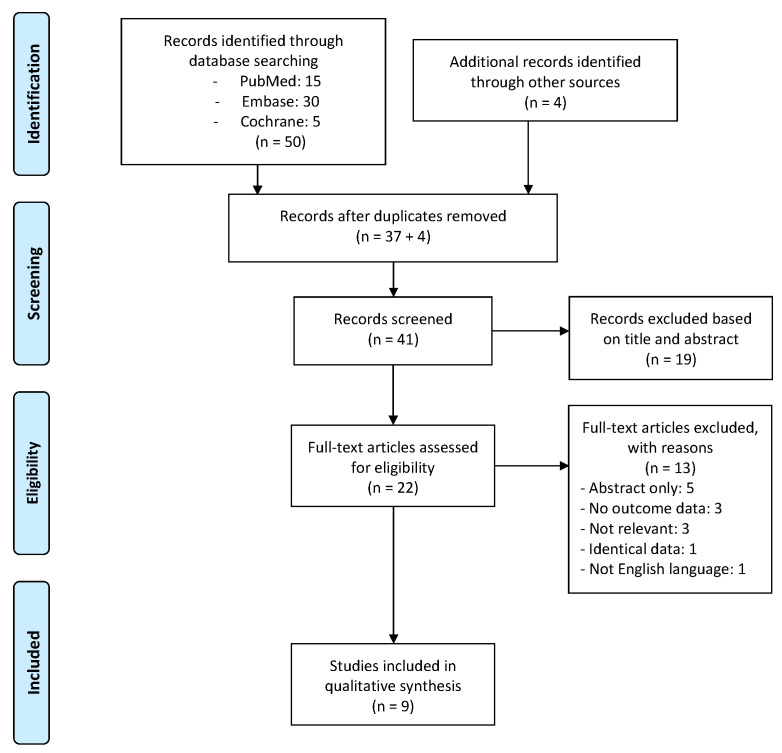
PRISMA flow of studies included in our review.

A total of 54 articles were identified through electronic database searches and assessed for eligibility, with 41 remaining following removal of duplicates. We removed 19 papers following abstract review (four review articles and 15 not related to UAE), with eligible studies remaining for full-text review. A further 13 were removed on full-text review as five studies had no full texts available (conference abstracts), three had no outcome data related to UAE safety, incidence of PES or the aetiological basis of the complication. Nine studies remained for final analysis after full text assessment [3,8,9,10,18,19,20,21,22]. The screening process is described in Figure 1.

### 3.2. Study Characteristics

The salient characteristics of included papers are described in Table 1 and Table 2. Observational studies comprised the majority of relevant studies (6/9), with 5/9 retrospective in design. The rate of PES was documented in 5/8 studies (excluding case report) ranging from 4–34.6%. 5/9 studies suggest an inflammatory aetiology of PES.

### 3.3. Quality Assessment

EPHPP quality assessment tool was used to analyse each included study. Of the seven included studies, one defined as “strong”, five as “moderate” and three as weak.

### 3.4. Characteristics of Fibroids from Studies

The salient characteristics of fibroids identified in included papers are described in Table 3. The major type of fibroid was stated in 4/9 studies. The mean fibroid size was listed in 6/9 studies.

## 4. Discussion

### 4.1. Aetiology and Potential Biomarkers

The aetiology of post-embolisation syndrome (PES) following uterine artery embolization (UAE) remains incompletely understood. It has been postulated to result from the release of degradation products, inflammatory and vasoactive mediators including interleukin-6 and tumour necrosis factor- α following necrosis and infarction of tissue [10,19]. The release of ischaemic fibroid products into the vascular system can result in a swinging fever, pelvic or abdominal pain plus or minus nausea and vomiting. This is commonly followed by leucopenia, leukocytosis and possible increased C-reactive protein. Further clinical studies are necessary to characterize the specific underlying mechanism pertaining to the syndrome [21,23]. Despite a paucity of studies concerning the specifics of the mechanism underlying PES, the current literature provides several observations that support the hypothesis of an inflammatory aetiology [19,20]. Additionally, previous literature has demonstrated that pain level correlates with volume and percentage of ischaemic myometrial tissue, suggesting a potential relationship between fibroid size and the severity of PES symptoms.

Sabre et al. assessed the predictive values of neutrophils, lymphocytes, platelets, neutrophils to lymphocytes ratio (NLR) and platelets to lymphocytes ratio (PLR) to highlight the occurrence of PES after UAE [18]. Sixty-two patients were recruited, of whom 19.4% (12/62) developed PES. The researchers reported a statistically significant elevation in platelets in the PES group (*p* = 0.036), concluding that platelet levels may be significant when it comes to predicting adverse outcomes [18]. Futhermore, Ganguli et al. investigated the association of leucocytosis as a component of PES in the absence of infectious aetiology. They reported an incidence of leucocytosis in 21% of patients with PES beyond the over the first day post-procedure [21]. This is a challenge in diagnosing PES, as it must be differentiated from more sinister causes such as bacterial sepsis and tumour lysis syndrome.

Magnetic resonance imaging has been utilised to assess uterine ischemia after UAE. Ruuskanen et al. aimed to evaluate the role of myometrial and fibroid ischemia in the pathogenesis of the pain experienced after UAE. They determined that patients who suffered a high level of pain tended to have moderate to severe myometrial ischaemia, however the extent of fibroid ischaemia did not correlate with patient experienced pain [22]. UAE of large fibroids was previously reported to increase the risk of the patient suffering PES [24]. However, Prollius et al. highlighted contradictory findings in a study of 61 women, 12 with a uterus greater than 780 cm^3^ and 49 with a uterus less than 780 cm^3^ as measured on ultrasound. They reported comparable efficacy and complications including incidence of PES [9].

### 4.2. Perioperative Management

The peri-procedural medical management for UAE is highly variable. Bilhim et al. supported an inflammatory origin for PES by evaluating the utilization of non-steroidal anti-inflammatory drugs (NSAIDS) in the management of PES symptoms. In this study, they administered NSAIDs pre-procedure, during the procedure and post UAE via a variety of routes (oral, intravenous or intra-arterially) [19]. Overall the researchers concluded that the utilization of NSAIDs during UAE reduced patient reported symptomatology of PES including abdominal discomfort and pyrexia [19]. In addition, at the same institution Pisco et al. evaluated whether UAE could be safely performed as an outpatient procedure, evaluating 234 patients who underwent UAE [20]. The included patients commenced treatment with a NSAID, an acid-suppressing drug, an anti-histamine, and a laxative one day prior to their procedure. Post-procedural pain scores were assessed at several intervals including the evening of UAE, prior to hospital discharge and following discharge. The mean pre-procedure pain scores were 0.9, increasing to 2.5 four to eight hours post-procedure, before falling to 0.9 at discharge. In this study, the entire cohort were discharged within eight hours following embolisation. Bilhim et al. concluded that UAE can be performed safely as an outpatient procedure and utilization of a peri-procedural medication regimen including a NSAID can significantly reduce patient post-procedural pain [20]. These two studies may also serve to support further an inflammatory aetiology [19,20].

Several studies report rapid recovery following PES post UAE, in addition to shortened hospital stay compared to hysterectomy [3,8]. The REST study group noted a mean hospital stay reduction of four days compared to surgery, although 3/106 patients required further admissions over the post-procedure year [3].

### 4.3. Clinical Significance

PES is a frequently occuring sequelae of UAE. Data specifically related to the incidence and aetiology of PES post embolisation of the uterine artery is limited. Overall the incidence of PES was found to range from 19.4% to 34.5%, which was similar to previously reported PES rate following embolisation of the prostatic artery [23]. The heterogenicity between the studies made the rate difficult to interpret. We believe, that this rate is likely to be underestimated given the overlap of symptoms with alterative diagnoses such as in infection. Additionally, the syndrome can occur up to one week post-procedure, thus patient may be discharge making recognition more difficult.

### 4.4. Strengths and Limitations of the Study

The primary strength of the present study is that independent screening and abstraction for was performed, resulting in the largest systematic review on the topic to our knowledge. However, there were several limitations including small study number of studies available on the topic of PES specific to UAE, with only five studies referring to the potential aetiological basis of the syndrome and six related to the incidence of the syndrome. Furthermore, there was heterogeneity in reporting of study aims and outcomes as exist with all systematic reviews. Three studies do not report embolic agent utilised, whilst the remaining six used a variety of agents. Two prospective clinical trials were identified, but neither aimed to assessed specifically the aetiological basis of PES.

## 5. Conclusions

PES is the most common adverse event and frequent indication for hospitalization following UAE, yet it is a poorly understood phenomenon with a relative paucity of literature to date. The systematic review highlighted limited studies, whilst there was heterogeneity of reporting outcomes, including those related to aetiology and incidence of PES. Further research is necessary to advance our understanding of PES to define the biological basis of the syndrome with more certainty and consensus on peri-procedure management to reduce incidence and improve patient outcomes.

## Figures and Tables

**Table 1:** Demographic information of included studies.

Author	Year	Country	Study Type	Number of Studies
Sabre [18]	2021	USA	Retrospective, Observational	62
Prollius [9]	2004	S. Africa	Prospective Case-control	63
Bilhim [19]	2010	Portugal	Retrospective, Observational	900
Leonhardt [10]	2004	Sweden	Retrospective, Observational (Case Report)	1
Salehi [8]	2015	Iran	Prospective, Observational	65
REST [3]	2007	UK	Prospective RCT	157 (106 UAE group)
Pisco [20]	2009	Portugal	Retrospective, observational	234
Ganguli [21]	2008	USA	Retrospective, observational	78
Ruuskanen [22]	2009	Finland	Prospective Clinical trial	62

**Table 2:** Aims, results, incidence and aetiology of PES of included studies.

Author	Aim	Results	Embolisation Substance	PES Rate, n (%)	Aetiological Basis PES	Assessment Grade
Sabre [18]	Assess predictive value of neutrophils, lymphocytes, platelets, neutrophils to lymphocytes ratio (NLR), platelets to lymphocytes ratio (PLR) in identifying the occurrence of PES post UAE	Patients with a preprocedural platelet count less than 336 × 10^3^/uL were less likely to have PES	Not specified	12/62 (19.4)	Inflammatory	Moderate
Prollius [9]	Compare the efficacy of UAE for large versus standard fibroids	Large uterus does not decrease UAE’s efficacy	Polyvinyl alcohol (2–300 mg)	18/51 (34.6) in in standard group, 3/12 (25.0) in large fibroids	None documented	Weak
Bilhim [19]	Assess NSAIDs in the management of the post-embolization symptoms	NSAID enables good control of the post-embolization symptoms	Non-spherical polyvnyl alcohol (dose unknown)	None documented	Inflammatory	Weak
Leonhardt [10]	Case report of common and expected reactions such as PES	PES on day 1 post-op, resolved by day 2	Polyvinyl alcohol (355–500 μm)	1/1 (100.0)	Inflammatory	Weak
Salehi [8]	Evaluate the efficacy and complications of UAE in patients with symptomatic uterine fibroids	UAE was a successful treatment for uterine fibroids that preserved the uterus, had minimal complications, and required short hospitalization and recovery	Polyvinyl alcoholol with or without gel foam (88.7%), Embo-Gold (8.1%) and Embosphere (3.2%) (dose unknown)	5/65 (7.7)	None documented	Moderate
REST [3]	Compare UAE to surgery in women with symptomatic uterine fibroids. Assess QoL after 1 year	Comparable QoL between groups. Faster recovery time in UAE group (1v 5 days). Major complication one year post UAE requiring further intervention 3/106	Not specified	26/106 (24.5)	None documented	Strong
Pisco [20]	Evaluated safety of UAE as an outpatient procedure	Pre-operative medication commenced on the day before UAE allow to be performed safely as an outpatient	Non-spherical polyvinyl alcohol particles (500–700 μm)	None documented	None documented	Moderate
Ganguli [21]	To assess for association between leucocytosis and PES	Clinically defined leukocytosis presented in 21% of all patients undergoing elective UAE first day post procedure	Not specified	16/65 (21)	Inflammatory	Moderate
Ruuskanen [22]	Evaluate uterine ischaemia after uterine artery embolisation (UAE) using magnetic resonance imaging	Selective embolisation may result in a therapeutic effect with fewer side effects	EmboSphere (550–700 μm)	None documented	Inflammatory	Moderate

**Table 3:** Characteristics of fibroids.

Author	Type (%)	Mean Size (cm^3^)
Sabre [18]	Intramural (30.6); subserosal (16.1;) submucosal (16.1); mixed (37.1)	414.7 (PES+) 904.8 (PES−)
Prollius [9]	Not specified	Unknown (>780 (*n* = 12) <780 (*n* = 49))
Bilhim [19]	Not specified	Unknown
Leonhardt [10]	Submucosal	86.63
Salehi [8]	Intramural (80.6); Submucosal (14.5); Subserosal (4.8)	Unknown
REST [3]	Not specified	579
Pisco [20]	Intramural (76.1); Submucosal (10.7); Subserosal (13.2)	110.5
Ganguli [21]	Not Specified	Unknown
Ruuskanen [22]	Not specified	200

## Data Availability

Not applicable.

## Supplementary Materials

The following supporting information can be downloaded at: https://www.mdpi.com/article/10.3390/diagnostics12122916/s1, Table S1: Study Protocol.

## Abbreviations

EPHPP—Effective Public Health Practice Project; NSAID—Non-steroidal anti-inflammatory drug; NLR—neutrophils to lymphocytes ratio; PLR—platelets to lymphocytes ratio; PES: Post-embolisation syndrome; QoL: quality of life; RCT—randomized controlled trial; UAE: uterine artery embolization.

## Appendix A. Electronic Database Search Terms

MEDLINE (Pubmed)

((post embolisation syndrome) OR (post embolisation syndrome)) AND ((uterine artery[Title/Abstract]) OR (uterine fibroid[Title/Abstract]))

EMBASE

(‘post embolization syndrome’/exp OR ‘post embolization syndrome’) AND (‘uterine artery’:ab,ti OR ‘uterine fibroid’:ab,ti)

Cochrane

(“post-embolisation syndrome”) AND ((“uterine artery”) OR (“Uterine fibroid”))
