# Supplementary material for: Uterine Artery Embolisation of Fibroids and the Phenomenon of Post-Embolisation Syndrome: A Systematic Review
Source: Diagnostics (Basel). 2022 Nov 23;12(12):2916. doi: 10.3390/diagnostics12122916 (PMC9776929; doi:10.3390/diagnostics12122916)
Supplement: Supplementary file 1 [file diagnostics-12-02916-s001.zip › diagnostics-2000939-supplementary.pdf]

Supplementary Table S1: Study Protocol.

TITLE	CHECKLIST ITEM	REPORTED ON PAGE #
1. Title:	Title<br>Uterine Artery Embolisation of Fibroids and the phenomenon of Post-Embolisation Syndrome: a systematic review<br>Registration<br>PROSPERO: CRD42022365162<br>https://www.crd.york.ac.uk/prospero/	
**BACKGROUND**		
2. Objectives:	The research question including components such as participants, interventions, comparators, and outcomes.<br><br>**Objective**<br>Systematic review of the literature to assess regarding the aetiology and management the phenomenon of post-embolisation syndrome following uterine artery embolization	
**METHODS**		
3. Eligibility criteria:	Study and report characteristics used as criteria for inclusion.<br><br>**Types of Studies**<br>Randomised controlled trials.<br>Observational studies.<br>Any measure of outcome.<br><br>**Types of participants**<br>Human	

	Unrestricted age, condition, surgery type.<br><br>**Inclusion Criteria**<br>Published studies (MEDLINE, EMBASE, Cochrane)<br>Published any time before October 2022.<br>English Language.		
4. Information sources:	Key databases searched and search dates.<br>**Electronic Searches**<br>MEDLINE (PUBMED)<br>EMBASE<br>Cochrane Central Register of Controlled Trials (CENTRAL)<br><br>**Search Date**<br>13/10/22<br><br>**Other Searches**<br>In addition, hand searches will be conducted of the reference lists of all articles retrieved to identify other potentially eligible articles.<br><br>**Search Strategy**<br><br>Pubmed/MEDLINE<br>((post embolisation syndrome) OR (post embolisation syndrome)) AND ((uterine artery[Title/Abstract]) OR (uterine fibroid[Title/Abstract]))<br>MeSH Terms (MEDLINE)<br>EMBASE (EMTREE)		

	**<u>Strategies to Minimize Bias</u>**<br>Data extracted by two reviewer with a third to assess where undecided<br>The Effective Public Health Practice Project (EPHPP) quality assessment tool was utilised to analysis quality of included studies. Quality areas assessed included study design, bias and study blinding, confounder management, data collection methods, intervention integrity and analysis. The EPHPP tool was used to grade each component, with each study then categorised as strong, moderate, or weak quality.<br>No meta-analysis planned<br><br>**<u>Data extraction</u>**<br>Two Reviewers to review independently<br>1. Michael Waldron<br>2. Owen O’Connor<br><br>**<u>Extraction Variables</u>**<br>1. Year of publication<br>2. Journal<br>3. Study design<br>4. Quality of study (Jadad and MINORS score)<br>5. Condition<br>6. Outcome<br>7. Intervention<br>8. Number of participants<br>9. Country of trial	
5. Risk of bias:	Methods of assessing risk of bias.<br>Effective Public Health Practice Project (EPHPP) quality assessment tool	
**RESULTS**		

6. Included studies:	Number and type of included studies and participants and relevant characteristics of studies.<br>Search yielded 54. 22 following abstract review. 9 remained for inclusion following review of full texts<br><br>Study Selection<br>A total of 54 articles were identified through electronic database searches and assessed for eligibility. The screening process is described in Figure 1. Twenty-two studies were eligible following review of abstracts, with nine remaining for final analysis after full text assessment.<br><br>Study Characteristics<br>The salient characteristics of included papers are described in table 1 and 2. Observational studies comprised the majority of relevant studies (6/9), with 5/9 retrospective in design. The rate of PES was documented in 5/8 studies (excluding case report) ranging from 4-34.6%. 5/9 studies suggest an inflammatory aetiology of PES.<br><br>Quality Assessment<br>EPHPP quality assessment tool was used to analyse each included study. Of the seven included studies, one defined as "strong", four as "moderate" and four as weak.	
7. Synthesis of results:		
8. Description of the effect:		
**DISCUSSION**		
9. Strengths and Limitations of evidence:	Brief summary of strengths and limitations of evidence (e.g. inconsistency, imprecision, indirectness, or risk of bias, other supporting or conflicting evidence)<br>The primary strength of the present study is that independent screening and abstraction for was performed, resulting<br><br>in the largest systematic review on the topic to our knowledge. However, there were several limitations including	

	small study and publication bias. Furthermore, the heterogeneity in reporting of study outcomes as exist with all systematic reviews. Additionally, the paucity of literature related to PES.	
10. Interpretation:	General interpretation of the results and important implications<br>PES is the most common adverse event and frequent indication for hospitalization following UAE, yet it is a poorly understood phenomenon with a relative paucity of literature to date. Further research is necessary to advance our understanding of PES to define the biological basis of the syndrome with more certainty and consensus on peri-procedure management to reduce incidence and improve patient outcomes.	
